# A Case of Delirium Tremens Treated with Large Doses of Tinct. Digitalis

**Published:** 1865-01

**Authors:** S. Barrett

**Affiliations:** Le Roy, Genesee county, N. Y.


ART. IV.—*A Case of Delirium Tremens treated with large doses of Tinct. Digitalis.* By S. BARRETT, M. D., of Le Roy, Genesee county, N. Y.

The attention of the medical profession the past few years has been directed to the treatment of delirium tremens with large doses of the tinct. of digitalis. While some have set their seal of condemnation upon it at once, others, as teachers, have raised their voice against it, assuming that it is an uncertain and dangerous remedy, especially when given in doses above that prescribed by the books, forgetting, as it seems to me, that we have in these cases a gastritis with a peculiar state of the nervous system to treat, and that it is the effect, and not the dose of the remedies we use, that we are to regard. Believing as I do that a few facts are of more value than many theories, and that this is one of the remedies that is still on trial, I submit the following case:

On the 22d of July last, I was called to see Dr. S. R., aged 35, of Stafford. He has drank to excess for several years—has hardly been sober for the past four or five years. I found him with all those hallucinations incident to such cases; had not slept for two nights or days; been taking opiates, morphine, and hyoscyamus with camphor, and some stimulants; pulse 130 per minute, weak and tremulous; great thirst and constant restlessness, eyes staring, incoherent talking, and constant effort to divest himself of all clothing; I attempted to quiet him by giving him chloroform, but it threw him into spasms, with very difficult breathing. When he came fully under its influence, he turned black in the face and ceased breathing, the heart ceased to beat, and he had every appearance of being dead. I turned him on his right side and kept up artificial respiration for some minutes, when the action of the heart commenced, and he soon began to catch for breath, and in a little time revived, but as delirious as ever. I then gave him ice to eat pretty freely and tinct. digitalis ℥ ss, which he took readily, and ordered him to have the same dose every four hours until he became quiet and slept. I left him to return the next day.

23d. Visited him to-day at 12 M.; found the doctor quiet, "clothed and in his right mind;" he had slept about six hours, had taken jss ℥ of the digitalis; pulse normal, skin moist, thirst gone. From this time he convalesced rapidly. Six days after he called to see me; appetite good; says he had never felt better in his life.

The few cases of this disease which have come under my care the past two years have been treated with this remedy, and the results have been to me every way satisfactory. That it is a specific I do not claim, only that it is worthy of trial.

